# New paradigms in whole genome sequencing: from lab bench to cell phone

**DOI:** 10.1186/1755-8166-7-S1-I27

**Published:** 2014-01-21

**Authors:** Jonathan O’Halloran

**Affiliations:** 1 QuantuMDx GROUP LIMITED, Newcastle, UK

## 

The DNA sequencing & MDx fields have seen dramatic advances since the first draft of the human genome was published, with companies reporting ever faster and cheaper methods. However, despite the race to attain the $1000 genome producing a plethora of exciting technologies, capillary electrophoresis (CE) is still being routinely used for targeted clinical sequencing and expensive real time PCR devices are still the work-horse of the MDx laboratory. QuantuMDx is developing a portable, handheld DNA sequencing device for PGx and infectious disease applications, to provide an alternative to slow and relatively expensive CE DNA sequencing & expensive slow and lab based MDx & CDx.

